# Identification of Differential Genes of DNA Methylation Associated With Alzheimer’s Disease Based on Integrated Bioinformatics and Its Diagnostic Significance

**DOI:** 10.3389/fnagi.2022.884367

**Published:** 2022-05-09

**Authors:** Fan Chen, Na Wang, Xiaping He

**Affiliations:** School of Basic Medical Sciences, Dali University, Dali, China

**Keywords:** Alzheimer’s disease, differentially expressed genes (DEGs), hub genes, DNA methylation, bioinformatics

## Abstract

**Background:**

Alzheimer’s disease (AD) is a common neurodegenerative disease. The pathogenesis is complex and has not been clearly elucidated, and there is no effective treatment. Recent studies have demonstrated that DNA methylation is closely associated with the pathogenesis of AD, which sheds light on investigating potential biomarkers for the diagnosis of early AD and related possible therapeutic approaches.

**Methods:**

Alzheimer’s disease patients samples and healthy controls samples were collected from two datasets in the GEO database. Using LIMMA software package in R language to find differentially expressed genes (DEGs). Afterward, DEGs have been subjected to enrichment analysis of GO and KEGG pathways. The PPI networks and Hub genes were created and visualized based on the STRING database and Cytoscape. ROC curves were further constructed to analyze the accuracy of these genes for AD diagnosis.

**Results:**

Analysis of the GSE109887 and GSE97760 datasets showed 477 significant DEGs. GO and KEGG enrichment analysis showed terms related to biological processes related to these genes. The top ten Hub genes were found on the basis of the PPI network using the CytoHubba plugin, and the AUC areas of these top ranked genes were all greater than 0.7, showing satisfactory diagnostic accuracy.

**Conclusion:**

The study identified the top 10 Hub genes associated with AD-related DNA methylation, of which *RPSA, RPS23*, and *RPLP0* have high diagnostic accuracy and excellent AD biomarker potential.

## Introduction

Alzheimer’s disease is an age-related neurodegenerative disease that deteriorates with age and over time and often occurs in the elderly over 65 years. Currently, there are more than 55 million AD patients worldwide, and this number continues to grow, which represents a challenge to public health (Gauthier et al., 2021). According to data published by the World Health Organization (WHO) in 2020, AD and other dementias are the seventh greatest cause of mortality globally and one of the most socially costly chronic diseases (World Health Organization, 2021). As AD worsens, patients experience memory loss, mood swings, and even loss of self-care ability (Burns and Iliffe, 2009). It is predicted that the average life expectancy of patients with AD ranges from 3 to 9 years from diagnosis to further disease worsening (Querfurth and LaFerla, 2010; Todd et al., 2013). If diagnosed and intervened in a timely manner, the onset of AD will be delayed by 5–10 years (Ma et al., 2019). In the global pandemic of coronavirus disease 2019 (COVID-19), AD patients were reported to be more susceptible to coronavirus invasion (Mok et al., 2020), and for AD patients who have been infected with COVID-19, their mortality rate is significantly higher than that of healthy elderly (Hardan et al., 2021). Thus, the diagnosis of early AD can provide good help for both patient care and prognosis as well as global public health. Cognitive testing, magnetic resonance imaging (MRI), positron emission tomography (PET), and detection of cerebrospinal fluid (CSF) biomarkers are currently used clinically to diagnose AD, but according to *ALZHEIMER’S DISEASE INTERNATIONAL* (ADI) data, more than 75% of AD patients worldwide are not diagnosed, with 90% of patients in low and intermediate income (Nakamura et al., 2018; Sun et al., 2018; Gauthier et al., 2021; World Health Organization, 2021). Similarly, more than 77% of clinicians in an ADI and WHO survey intended to use new blood tests to improve the diagnostic accuracy of AD in clinical practice (Gauthier et al., 2021). Due to the ease of availability, high specificity, and economic advantages of blood marker performance, that is currently of great practical significance for exploring blood biomarkers for the diagnosis of AD.

In recent years, increasing studies have focused on AD-related DNA methylation, one of the key mechanisms of epigenetic research, which alters the expression of genes at the transcriptional level through the upregulation, downregulation, or silencing of genes without changing the DNA sequence (Mishra and Li, 2020; Sharma et al., 2020). DNA methylation regulates neuronal differentiation early in central nervous system development, whereas DNA methylation levels in the cerebral cortex change dynamically throughout life (Mohn et al., 2008). With the progression of aging, the degree of DNA methylation differences increases and can affect the function of learning and memory (Sharma et al., 2020; Li et al., 2021). Based on the specificity of different brain regions, neurons can express hypermethylation or hypomethylation at different sites during AD pathogenesis (Sharma et al., 2020). Hypomethylation of APP can lead to enhanced aggregation of amyloid plaques which is an important factor in AD pathogenesis (Gasparoni et al., 2018).

In addition to age, genes and family genetic history are also non-modifiable risk factors for AD. For AD cases other than early-onset familial AD (FEOAD), 70% of these risks can be attributed to genetic factors (Gatz et al., 2006; Ballard et al., 2011). Due to the continuous development and application of microarray and next-generation sequencing technology (NGS), genetic data-based research is growing, providing strong support for deciphering disease-related genetic factors (Mishra and Li, 2020). Some of these studies have achieved meaningful results for the diagnosis, prognosis and genetic analysis of AD based on genetic data. As early as 1993, Genome-wide association studies (GWAS) revealed APOE ε4 as the most important genetic risk factor for AD (Freudenberg-Hua et al., 2018). More recent risk gene loci including *ABCA7*, *ACE*, *ADAM10*, and *ADAM17* have also been reported (Pimenova et al., 2018). Similarly, NGS also identified risk genes associated with AD pathogenesis such as *NOTCH3*, *TREM2*, and *ARSA* (Patel et al., 2019). Some studies on methylation of these genes have demonstrated a close relationship with the development of AD. In AD patients, hypomethylation at the *TREM2* intron 1 CpG site results in higher expression of *TREM2* mRNA in leukocytes than in healthy controls (Ozaki et al., 2017). Although CpG methylation in *ABCA7* may have less of an impact on brain *ABCA7* mRNA expression (Yu et al., 2015), the CpG islands of *ABCA7* exhibit significant hypomethylation compared to healthy human brains (Humphries et al., 2015). In addition, 19 CpG island sites in the *ABCA7* locus have been shown to be positively correlated with pathological AD diagnosis (Yu et al., 2015), including 12 CpG sites associated with Aβ content and 18 CpG sites associated with Tau tangle tightness (De Roeck et al., 2019). These results provide evidence that AD pathogenesis may involve methylation of associated risk genes. Since DNA methylation levels are more stable than mRNA levels (Paziewska et al., 2014), studies based on blood DNA methylation levels may provide insights into important biological pathways in AD development.

Therefore, in our study, differentially expressed genes (DEGs) correlated with DNA methylation in AD were analyzed in order to find blood biomarkers that can be used to diagnose early AD with potential therapeutic targets. The gene expression profiling data for GSE109887 and GSE97760 were obtained from the Gene Expression Omnibus (GEO) database. To elucidate the pathomechanism of AD-related DNA methylation, DEGs were subjected to enrichment analysis of the Gene Ontology (GO) and Kyoto Encyclopedia of Genes and Genomes (KEGG) pathways. We also used DEGs for constructing coexpression networks to obtain Hub genes with DNA methylation associated with AD, hoping to provide new directions and strategies for the diagnose and therapy of AD.

## Materials and Methods

### Collection of Gene Expression Profiling Data

Gene Expression Omnibus is a public genetic and genomic data source that accepts array- and sequence-based data submissions, and enables users to search and download experimental gene expression profiles (Edgar et al., 2002). GSE109887, a dataset containing data on DNA methylation and hydroxymethylation levels in the blood and middle temporal gyrus (MTG) regions of the brain in AD patients, was used to obtain DNA methylation levels of DEGs. The differential expression levels of DNA methylation vary in different brain regions and show an increase throughout the brain region with aging. Compared with other brain regions, the MTG shows a more significant difference in DNA methylation level (Coppieters et al., 2014). And according to research on AD patients AD-related pathological changes appeared first in the MTG regions (Ray and Zhang, 2010). In addition, the trend of differential DNA methylation levels in MTG regions of the brain was reported to be similar to that in blood (Lardenoije et al., 2019). To further investigate the effects of DNA methylation on the expression of genes, GSE97760 dataset was used to obtain the expression levels of DEGs in the blood of AD patients and normal controls, which then was used to compare the DNA methylation levels of DEGs obtained from the GSE109887 dataset.

The GSE109887 dataset was examined using the GPL10904 (Illumina HumanHT-12 V4.0 expression beadchip) platform and contained 78 samples derived from the MTG of the brain, of which 32 samples were derived from healthy individuals and 46 samples were derived from AD patients. The GSE97760 dataset was tested using the GPL16699 (Agilent-039494 SurePrint G3 Human GEv2 8 × 60K Microarray 039381) platform and contained 19 blood samples, 10 samples from healthy individuals and 9 samples from AD patients.

### Data Processing and Identification of Differentially Expressed Genes

Limma (linear models for microarray data, doi: 10.1093/nar/gkv00), is a generalized linear model-based differential expression screening method. We used the R package Limma (version 3.40.6) to perform differential analysis of downloaded expression profile datasets to obtain differential genes between normal and AD groups. Specifically, we first set the *P* value threshold to 0.01, | log2FC | to 1.25, then log2 transform the data, further using Bayes’ function to compute moderated t-statistics, moderated F-statistic, and log-odds of differential empirical Bayes moderation values of the standard common toward expression errors, and finally obtain the differential significance of each gene.

### Weighted Gene Coexpression Network Analysis

Weighted gene coexpression network analysis can identify gene sets with highly synergistic changes to recognize potential biomarker genes or therapeutic targets based on the correlation of phenotypes of gene sets (Wisniewski et al., 2013). Specifically, we calculated the Median Absolute Deviation (MAD) for each gene separately based on the previously downloaded gene expression profiling data and then eliminated the top 95% of the genes with the smallest MAD. Scale-free co-expression networks were further constructed by removing outlying genes and samples using the Good Samples expression method of the R software package WGCNA. Afterward, we analyzed all genes using Pearson’s correlation matrix and average linkage. The power function amn = | Cmn | ^β (set the soft threshold β to 6) is used to transform the adjacency matrix into a topological overlap matrix (TOM), and the corresponding difference (1-TOM) is calculated. To classify genes with similar expression profiles as gene modules, we performed hierarchical clustering based on the difference metric of TOM with at least 30 genes per module of the gene dendrogram. To further analyze the modules, we calculated the difference of module eigengenes, merging modules with a distance less than 0.25.

### Enrichment Analysis of Differentially Expressed Genes

We performed enrichment analysis using the R package clusterProfiler (version 4.2.1) to obtain results for gene set enrichment. *P*-values less than 0.05 were judged to be statistically significant. The obtained results have been visualized using R and Enrichr.

### Protein-Protein Interaction Network Analysis of Differentially Expressed Genes

We created the PPI network through the STRING database (Szklarczyk et al., 2021). Specifically, the results obtained in the STRING database used a total score greater than 0.4 as a requirement to obtain AD-related DNA methylation key genes. The final results have been visualized using Cytoscape software (version 3.7.0) (Shannon et al., 2003).

### Acquisition of Hub Genes

We used the plugin CytoHubba in Cytoscape to find the key genes in the PPI network (Shannon et al., 2003). Since the degree value of a protein correlates with the importance of a gene and proteins with higher degrees values are more likely to be key proteins (Chin et al., 2014), the algorithm of Degree was employed in our study to assess those genes. Obtained the top ten ranked genes as hub genes using the Degree algorithm.

### Establishment and Analysis of the Receiver Operating Characteristic Curve

We constructed receiver operating characteristic (ROC) curves based on the pROC package in R software and further assessed the value of Hub genes in the GSE109887 dataset for the early diagnosis of AD by calculating the area under the ROC curve (AUC). The Hub gene was considered to have good diagnostic accuracy when the AUC value was greater than 0.6. The results were visualized using the ggplot package.

## Results

### Differentially Expressed Genes

**FIGURE 1:**
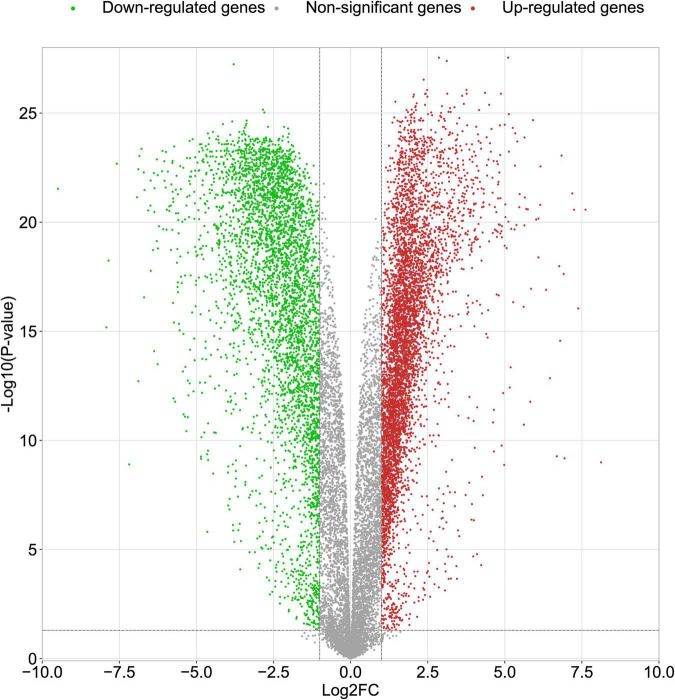
Volcano plots of DEGs in the GSE109887 datasets. The abscissa indicates fold change (Log2FC). The ordinate indicates –log10 (*p* value). Red dots indicate upregulated genes. Green dots indicate downregulated genes. Gray dots indicate non-significant genes.

**FIGURE 2:**
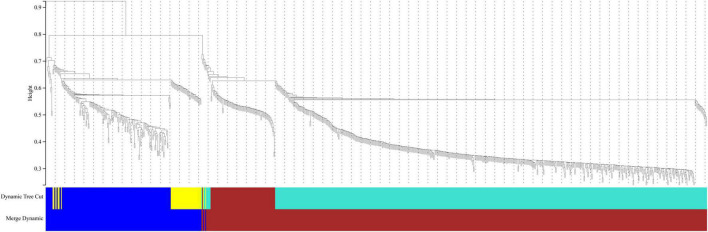
Cluster dendrogram of genes in the GSE109887 datasets. Gene dissimilarity based on 1-TOM. Different colors represent different modules of gene clustering.

**FIGURE 3:**
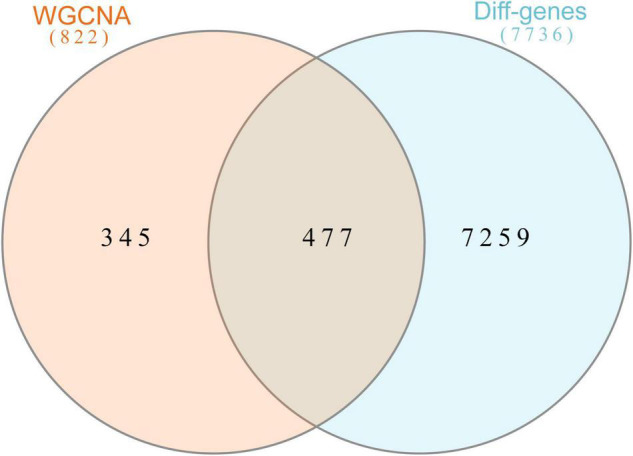
Overlapping genes in WGCNA versus DEGs were analyzed with Venn. Orange circle represents genes in WGCNA. Blue circle represents differential genes.

**FIGURE 4:**
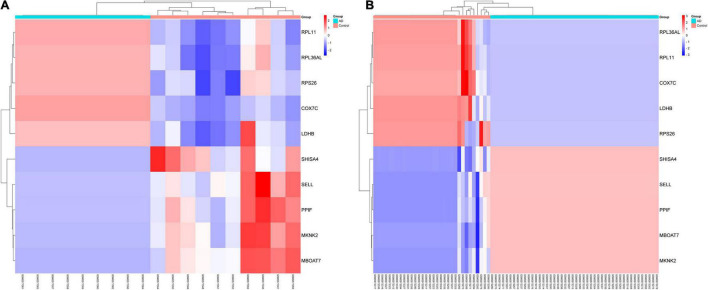
By analyzing two datasets, GSE97760 and GSE109887, a cluster heatmap of the top 10 DEGs between AD and normal controls was obtained. **(A)** Expression levels of DEGs. **(B)** DNA methylation of DEGs.

In this study, we compared two datasets, GSE109887 and GSE97760, and analyzed differentially expressed genes in a total of 42 normal control samples and 55 AD patient samples using the Limma package in R software. In the GSE109887 dataset, according to the set criteria (*P* < 0.01, | log2FC| = 1.25), we obtained 7,736 DEGs, including 4,230 upregulated DEGs and 3,506 downregulated DEGs (Figure 1). By using WGCNA analysis, we obtained two enriched modules containing 194 upregulated genes and 628 downregulated genes (Figure 2), which were intersected with DEGs after limma analysis, respectively, to obtain the final 477 DEGs (including 131 upregulated genes and 346 downregulated genes) (Figure 3). In addition, we analyzed the expression levels of the top 10 DEGs in the blood of the GSE97760 dataset. Figures 4A,B are the cluster analysis of the top 10 DEGs (differential expression level and DNA methylation level, respectively). Table 1 lists the basic information of the differential expression level and DNA methylation level of the top 10 DEGs.

**TABLE 1:** Differential expression level and DNA methylation level of the top 10 DEGs.

Up-regulated genes	Description	LogFC (DNA methylation)	LogFC (expression level)
RPL36AL	Ribosomal protein L36a like	8.12	−1.15
COX7C	Cytochrome Coxidase subunit 7C	7.19	−3.93
LDHB	Lactate dehydrogenase B	4.41	−1.74
RPS26	Ribosomal protein S26	4.13	−1.58
RPL11	Ribosomal protein L11	3.95	−1.94
**Down-regulated genes**	**Description**	**LogFC (DNA methylation)**	**LogFC (expression level)**
SELL	Selectin L	−6.71	7.96
SHISA4	Shisa family member 4	−6.56	8.42
MBOAT7	Membrane bound O-Acyltransferase domain containing 7	−6.41	8.69
PPIF	Peptidylprolyl isomerase F	−6.38	7.91
MKNK2	MAPK interacting serine/threonine kinase 2	−6.36	8.42

*LogFC, log2 fold change.*

### Gene Ontology and Pathway Enrichment Analysis of Differentially Expressed Genes

**FIGURE 5:**
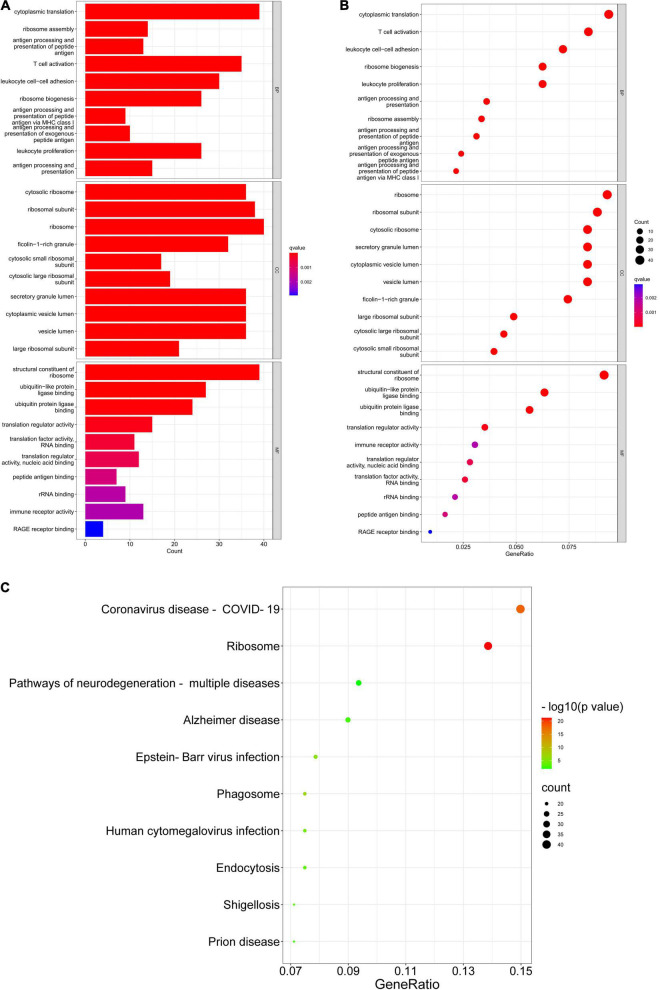
GO enrichment and KEGG pathway. **(A,B)** GO enrichment analysis of DEGs. **(C)** KEGG pathway analysis of DEGs.

Gene Ontology (GO) consists of three aspects in the biological field: molecular function, cellular composition, and biological process (Ashburner et al., 2000; Gene Ontology Consortium, 2021). The Kyoto Encyclopedia of Genes and Genomes (KEGG) is a database that understands the advanced functions and utilities of biological systems (Kanehisa et al., 2022). Mastering the potential biological functions of genes can help to explore the role of DEGs in the pathogenesis of AD. Based on this, we performed enrichment analysis of DEGs in DNA methylation associated with AD. The results of GO analysis are presented in Table 2, which shows that these DEGs are significantly enriched in functions related to ribosomes and cytoplasm, involving biological processes including cytoplasmic translation, T cell activation, proliferation and cell-cell adhesion of leukocytes, and ribosomal biological processes (Figures 5A,B). KEGG pathway enrichment analysis indicated that these genes were significantly enriched in Coronavirus disease-COVID-19, Ribosome, Pathways of neurodegeneration-multiple diseases, Alzheimer’s disease, and Epstein-Barr virus infection (Figure 5C). Table 3 presents information on these pathways.

**TABLE 2:** The Gene Ontology (GO) terms for the DEGs.

GO ID	GO term	Counts	*p* value	*q* value
**GOTERM_BP**				
GO:0002181	Cytoplasmic translation	39	5.45E-31	1.72E-27
GO:0042255	Ribosome assembly	14	5.01E-11	7.90E-08
GO:0048002	Antigen processing and presentation of peptide antigen	13	8.26E-10	7.55E-07
GO:0042110	T cell activation	35	1.14E-09	7.55E-07
GO:0007159	Leukocyte cell-cell adhesion	30	1.20E-09	7.55E-07
GO:0042254	Ribosome biogenesis	26	3.46E-09	1.82E-06
GO:0002474	Antigen processing and presentation of peptide antigen *via* MHC class I	9	5.75E-09	2.59E-06
GO:0002478	Antigen processing and presentation of exogenous peptide antigen	10	7.14E-09	2.82E-06
GO:0070661	Leukocyte proliferation	26	1.25E-08	4.00E-06
GO:0019882	Antigen processing and presentation	15	1.27E-08	4.00E-06
**GOTERM_CC**				
GO:0022626	Cytosolic ribosome	36	6.17E-34	2.08E-31
GO:0044391	Ribosomal subunit	38	1.67E-26	2.82E-24
GO:0005840	Ribosome	40	9.57E-24	1.07E-21
GO:0101002	Ficolin-1-rich granule	32	1.03E-19	8.70E-18
GO:0022627	Cytosolic small ribosomal subunit	17	7.42E-18	5.00E-16
GO:0022625	Cytosolic large ribosomal subunit	19	9.27E-18	5.21E-16
GO:0034774	Secretory granule lumen	36	1.09E-15	5.23E-14
GO:0060205	Cytoplasmic vesicle lumen	36	1.46E-15	6.14E-14
GO:0031983	Vesicle lumen	36	1.77E-15	6.62E-14
GO:0015934	Large ribosomal subunit	21	5.20E-14	1.75E-12
**GOTERM_MF**				
GO:0003735	Structural constituent of ribosome	39	1.31E-26	7.13E-24
GO:0044389	Ubiquitin-like protein ligase binding	27	6.26E-09	1.71E-06
GO:0031625	Ubiquitin protein ligase binding	24	1.26E-07	2.30E-05
GO:0045182	Translation regulator activity	15	9.19E-07	0.000125311
GO:0008135	Translation factor activity, RNA binding	11	3.73E-06	0.000406315
GO:0090079	Translation regulator activity, nucleic acid binding	12	7.76E-06	0.000705088
GO:0042605	Peptide antigen binding	7	1.60E-05	0.001248783
GO:0019843	rRNA binding	9	2.62E-05	0.00178531
GO:0140375	Immune receptor activity	13	3.14E-05	0.001905303
GO:0050786	RAGE receptor binding	4	5.36E-05	0.002923918

*BP, biological process; CC, cellular composition; MF, molecular function.*

**TABLE 3:** The KEGG pathway for the DEGs.

ID	Pathway	Counts	*p* value	*q* value
hsa05171	Coronavirus disease – COVID-19	40	1.64E-18	1.84E-16
hsa03010	Ribosome	37	5.98E-22	1.34E-19
hsa05022	Pathways of neurodegeneration – multiple diseases	25	1.36E-02	0.086856153
hsa05010	Alzheimer’s disease	24	1.83E-03	0.025655536
hsa05169	Epstein-Barr virus infection	21	2.74E-06	0.000153521
hsa04145	Phagosome	20	1.07E-07	7.98E-06
hsa05163	Human cytomegalovirus infection	20	4.84E-05	0.001550828
hsa04144	Endocytosis	20	2.19E-04	0.004909197
hsa05131	Shigellosis	19	4.90E-04	0.009980422
hsa05020	Prion disease	19	1.63E-03	0.024347382

### The Protein-Protein Interaction Network and Hub Genes Analysis

**FIGURE 6:**
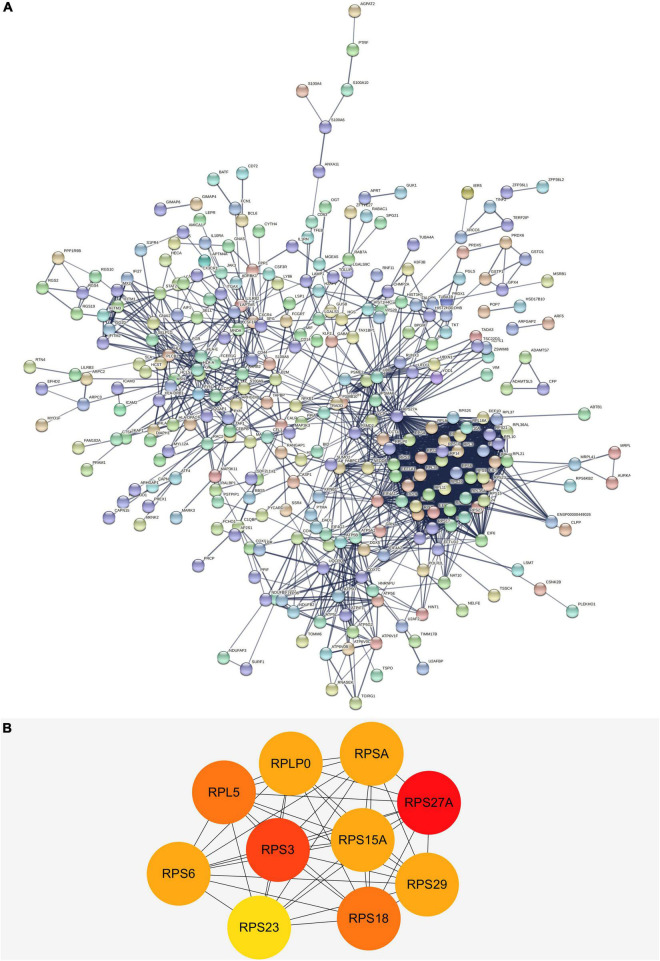
**(A)** The PPI network of DEGs in AD. There are a total of 308 nodes and 3,244 edges. Nodes represent proteins and edges represent interactions between proteins. **(B)** The top 10 hub genes with higher degree screened from a PPI network according to the Degree algorithm. Darker color indicates degree. PPI, protein-protein interaction; DEGs, differentially expressed genes.

We built a PPI network through the STRING database, and in addition to the nodes with interrupted networks, Figure 6A presents 308 nodes and 3,244 edges in the PPI network, where edges indicate interactions among genes. The top 10 Hub genes (*RPL5, RPLP0, RPS15A, RPS18, RPS23, RPS27A, RPS29, RPS3, RPS6*, and *RPSA*) were obtained based on Degree algorithm in CytoHubba (Figure 6B). Afterward we obtained the differential expression of these Hub genes without DNA methylation modification from the GSE97760 dataset and found that the expression of these genes was downregulated, contrary to the level of DNA methylation. Information about these Hub genes is listed in Table 4.

**TABLE 4:** The information of top 10 hub genes.

No.	Name	Full name	Degree score	AUC	LogFC (DNA methylation)	LogFC (expression level)	Brief function
1	RPS27A	Ribosomal protein S27A	142	0.756	3.14	−2.81	Monomeric ubiquitin-ribosome fusion gene involved in encoding a fusion protein. It can promote the development of neurodegenerative diseases including AD (Khayer et al., 2020)
2	RPS3	Ribosomal protein S3	102	0.777	1.53	−2.18	It is involved in composing the eukaryotic ribosomal 40S subunit, regulating the initiation of ribosome maturation and translation with the eukaryotic initiation factors elF2 and elF3, and is involved in apoptosis (Kim et al., 2018)
3	RPL5	Ribosomal protein L5	98	0.779	2.41	−1.94	It is involved in encoding ribosomal proteins that catalyze protein synthesis and consists of a small 40S subunit and a large 60S subunit (Safran et al., 2010)
4	RPS18	Ribosomal protein S18	98	0.797	1.37	−1.89	It encodes a ribosomal protein that is a component of the 40S subunit (Safran et al., 2010)
5	RPS29	Ribosomal protein S29	96	0.776	1.43	−1.58	It encodes a ribosomal protein that is a component of the 40S subunit and a member of the S14P family of ribosomal proteins (Safran et al., 2010)
6	RPLP0	Ribosomal protein LP0	96	0.848	1.54	−1.65	An acidic ribosomal protein. It is involved in ERS and autophagy. It is associated with pathological Tau in AD (Ban et al., 2014; Artero-Castro et al., 2015; Evans et al., 2019)
7	RPSA	Ribosomal protein SA	96	0.831	1.43	−1.25	Components of the 40S ribosomal subunit. Also known as 37/67 kDa high-affinity laminin receptor. May contribute to AD by regulating the process of apoptosis (Da Costa Dias et al., 2013; Jovanovic et al., 2015)
8	RPS15A	Ribosomal protein S15A	96	0.776	2.44	−1.21	One of the subunits of the 40S ribosomal protein. It promotes glioma development (Zhang et al., 2016)
9	RPS6	Ribosomal protein S6	96	0.779	1.48	−0.90	It encodes a cytoplasmic ribosomal protein, which is a component of the 40S subunit. It is a major substrate of protein kinases in the ribosome (Safran et al., 2010)
10	RPS23	Ribosomal protein S23	94	0.814	2.90	−0.44	Highly conserved component of the 40S subunit in eukaryotes. To maintain the fidelity of protein translational synthesis. The RPS23RG family of pseudogenes generated by inversion is involved in the development of AD (Zhang et al., 2009; Huang et al., 2010; Zhou et al., 2015; Hipp et al., 2019; Zhao et al., 2019; Ma et al., 2020; Martinez-Miguel et al., 2021)

*AUC, area under the curve; logFC, log2 fold change.*

### Receiver Operating Characteristic Curve Analysis

**FIGURE 7:**
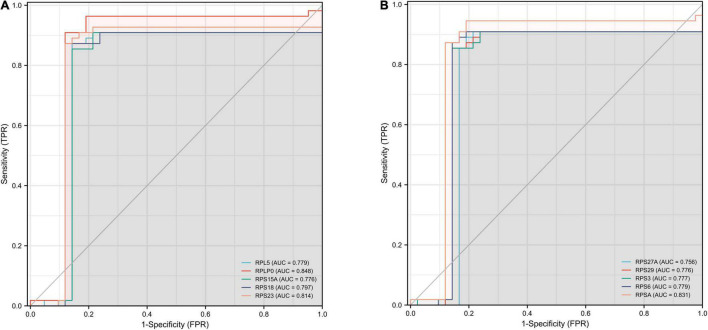
ROC curves for the top ten hub genes. The abscissa represents the FPR and the ordinate represents the TPR. The value of the AUC represents the accuracy of the diagnostic value, with values closer to 1 indicating better accuracy. ROC, operating receiver characteristic; FPR, false positive rate; TPR, true positive rate; AUC, area under the curve. **(A)** RPL5, RPLP0, RPS15A, RPS18, and RPS23. **(B)** RPS27A, RPS29, RPS3, RPS6, and RPSA.

We performed ROC curve analysis to validate the accuracy of Hub gene for the early diagnosis of AD. The closer the value of AUC is to 1, representing a greater diagnostic value. Our results showed that the top ten genes all had good diagnostic value. As shown in Figures 7A,B, the maximum AUC was 0.848 and the minimum AUC was 0.776. Among them, the AUC of *RPS23, RPSA*, and *RPLP0* was greater than 0.8, indicating that these three genes may play a good accuracy in the early diagnosis of AD pathogenesis.

## Discussion

Over the past century, people have not stopped exploring AD. Many hypotheses have attempted to elucidate the pathogenesis of AD, such as oxidative stress, neuronal excitotoxicity, and tau hyperphosphorylation (Ju and Tam, 2022). In recent years, it has been demonstrated that epigenetic mechanisms play a significant part in the formation and development of memory during growth under physiological and pathological conditions, and are likely to participate in the pathogenesis and progression of AD (Sananbenesi and Fischer, 2009; Fischer et al., 2010; Day and Sweatt, 2011). DNA methylation, as one of the epigenetically important mechanisms, has been demonstrated to have a place in AD pathogenesis (Mohn et al., 2008; Qazi et al., 2018; Sharma et al., 2020), and genes and proteins associated with DNA methylation are potential biomarkers in AD (Qazi et al., 2018). However, the specific mechanisms underlying need to be further explored.

DNA methylation is the process of adding a methyl group to DNA mediated by DNA methyltransferases (DNMTs). The process does not involve changes in DNA sequence, but rather influences DNA activity and work ability (Coppedè, 2021). It is required for neuronal differentiation, embryonic development and regulation of gene expression levels, and is also involved in regulation of biological processes such as brain development and memory formation (Jones, 2012). Impairment of DNA methylation has already been shown to have a relationship with neural development and neurodegenerative diseases, in studies in primates and rodents, lack of dietary B-vitamins or excessive exposure to metals (lead) early in development can affect DNA methylation levels *in vivo*, resulting in changes in Aβ peptide gene expression associated with the pathogenesis of AD, such as BACE1, PSEN1, and APP genes (Fuso et al., 2005, 2011; Bihaqi et al., 2011). Impaired expression and activity of enzymes involved in DNA methylation and demethylation can also be detected in the brains of these animals (Fuso et al., 2011). Alterations in genes associated with DNA methylation and Aβ can also be detected in the brains of AD patients after death (Wang et al., 2008). This is consistent with a previous study concluding that alterations in DNA methylation is one of the causes of decreased neural function in the brain during aging (Liu et al., 2009). Reduced DNA methylation levels in mitochondria-associated regions can also be detected in peripheral blood of patients with delayed AD (De Jager et al., 2014). A study has shown that increased DNA methylation levels of the brain-derived neurotrophic factor (BDNF) promoter result in reduced BDNF mRNA protein expression and add a higher risk of AD development (Xie et al., 2017). In terms of AD treatment, some progress and success has been made in adding DNA methyl donors (folic acid and vitamin B12) to the diet of AD patients (Durga et al., 2007; Haan et al., 2007).

Based on the close link between DNA methylation and the development of AD, we downloaded datasets related to DNA methylation from the GEO data repository for bioinformatics analysis to obtain key genes implicated in AD pathogenesis. In our work, the DEGs in the GSE109887 dataset were first screened and additionally the expression of these DEGs when not DNA methylated was analyzed from the GSE97760 dataset. Afterward, these genes were subjected to GO and KEGG enrichment analysis. The results of GO enrichment analysis showed that cytoplasmic translation, ribosome production and assembly, and ubiquitination of proteins were engaged in the production and progression of AD. This is in line with prior studies indicating that DNA methylation plays an essential role in the advancement of AD. This indicates that the results of our biological information analysis are acceptable.

In our work, the top 10 DEGs of DNA methylation associated with AD were selected: *RPL5, RPLP0, RPS15A, RPS18, RPS23, RPS27A, RPS29, RPS3, RPS6*, and *RPSA*. Through the analysis results of the ROC curve, *RPS23, RPSA*, and *RPLP0* all had very excellent diagnostic accuracy.

A recent experiment analyzing cell and molecular markers in donor brain samples by a combination of Fluoro deoxy glucose positron emission tomography (FDG-PET) and Allen Human Brain Atlas revealed that genes related to cytoplasmic ribosomes including *RPLP0* showed high enrichment (Patel et al., 2020). Notably, studies on the synthesis of ribosomes and proteins in the cytosol have been demonstrated many years ago to be associated with mild cognitive impairment and AD pathogenesis (Albert et al., 2011). These results are in high agreement with our analysis of DEGs that the top ranked Hub genes have surprisingly high correlations with ribosomal proteins and indicate that ribosomal protein genes associated with DNA methylation may be a new risk locus for AD. Ribosomes, as organelles that regulate intracellular protein biosynthesis and translation, are closely related to cell development and the lifespan of organisms (Nosrati et al., 2014). There is no doubt that AD itself, as a disease with Tau protein hyperphosphorylation and Aβ misfolding, is inseparable from abnormalities in intracellular biological processes related to protein synthesis and DNA transcription.

Additionally, we analyzed the expression levels of Hub genes by GSE97760 dataset (Table 4), consistent with previous findings that differential expression of DNA methylation can affect gene activity and is usually associated with repression of gene expression (Coppieters et al., 2014). *RPS23* is a highly conserved component of the 40S subunit in eukaryotes (Ma et al., 2020). It is involved in physiological and pathological processes such as tumorigenesis, immune signaling and growth and development (Zhou et al., 2015). Disturbed protein balance is a key factor leading to aging and age-related diseases, and translation is one of its key determinants (Hipp et al., 2019). And one of the most important roles of *RPS23* is to maintain the fidelity of protein translational synthesis. Recently, studies have been conducted to explore how the fidelity of protein synthesis can be regulated to prolong the life span of organisms starting from *RPS23* (Martinez-Miguel et al., 2021). Like genes encoding ribosomal proteins, *RPS23* has multiple processed pseudogenes dispersed in the genome, of which closely related to AD is the *RPS23RG* family produced by the inversion of *RPS23* (Huang et al., 2010). It has been shown that *RPS23RG1* and *RPS23RG2* can interact with adenylyl cyclase and upregulate cAMP levels, which further increases PKA activity, thereby limiting the activity of GSK-3, reducing Aβ production and tau hyperphosphorylation to resist the development of AD (Zhang et al., 2009; Hipp et al., 2019). Among them, *RPS23RG1* has also been shown to be a gene essential for maintaining synaptic integrity and resisting AD-related cognitive deficits (Zhao et al., 2019). In our study, it was found that the DNA methylation level of RPS23 was highly upregulated in AD patients, which may downregulate the gene expression (Table 4). Based on the important function of RPS23 and our results, we hypothesized that the expression of RPS23 can be downregulated by DNA methylation and thus participate in the development of AD.

*RPSA* is a non-integrin laminin receptor, also known as 37/67 kDa high-affinity laminin receptor precursor/laminin receptor (LRP/LR; Jovanovic et al., 2015). Studies have shown that knockdown of *RPSA* can lead to neurodegenerative diseases through apoptosis (Lowe and Lin, 2000), which is consistent with our findings that the expression of RPSA in AD patients was downregulated through DNA methylation (Table 4). While some other studies have pointed out new insights of *RPSA* in AD. Da Costa Dias et al. (2013) found that *RPSA* co-localizes with Aβ42 on the cell surface, and can physically bind to peptides that synthesize Aβ42, and low activity of *RPSA* can lead to enhanced Aβ42 toxicity. Research on targeting RPSA for prion diseases has been reported (Jovanovic et al., 2015), and the development of RPSA-targeted drugs can provide a new perspective for the treatment of AD.

*RPLP0* is an acidic ribosomal protein capable of mediating cell cycle arrest and apoptosis induced by phospholipase A and acyltransferase 4 (PLAAT4; Ban et al., 2014). It has been shown that *RPLP0* can interact with PLAAT4, and a significant reduction in cell survival has been observed in cells that overexpress PLAAT4 or knockdown RPLP0 (Wang et al., 2019). RPLP0 was essential for maintaining ribosome activity (Remacha et al., 1995), and downregulation of RPLP0 mRNA can mediate Endoplasmic reticulum stress response to induce abnormal autophagy (Artero-Castro et al., 2015). In K369I tau transgenic mice and rTg4510 tau transgenic mice, it was found that tau hyperphosphorylation resulted in decreased expression of RPLP0 (Evans et al., 2019). Similarly, in our study, the upregulated DNA methylation level of *RPLP0* in AD patients could lead to a decrease in the expression level of genes (Table 4).

Although our study found some genes with DNA methylation associated with AD development, and on this basis, the accuracy of Hub gene for the early diagnosis of AD was analyzed. However, the conclusions of our study still have some deficiencies. All of our research results are based on GEO public database and already published data, and due to sample size and platform limitations, further biological or clinical experiments are needed to confirm our conclusions in the future. But, in general, these hub genes provide new insights and directions for explaining AD pathogenesis and may become potential biomarkers and targeted therapies for accurate diagnosis and therapy of early AD in the near future.

## Conclusion

In summary, our study identified the top 10 hub genes associated with AD-related DNA methylation. These findings indicate that these genes may have participated in the pathological process of AD. Among them, *RPS23, RPSA*, and *RPLP0* have high diagnostic accuracy and excellent AD biomarker potential, which is worthy of further attention and research.

## Data Availability Statement

The datasets presented in this study can be found in online repositories. The names of the repository/repositories and accession number(s) can be found below: https://www.ncbi.nlm.nih.gov/, GSE109887; https://www.ncbi.nlm.nih.gov/, GSE97760.

## Author Contributions

XH provided the experimental ideas of this study and reviewed and revised the first draft of the manuscript. FC and NW did the data collection and verified by XH. FC did the data analysis, visualization of results, and wrote the first draft of the manuscript. All authors contributed to the article and approved the submitted version.

## Conflict of Interest

The authors declare that the research was conducted in the absence of any commercial or financial relationships that could be construed as a potential conflict of interest.

## Publisher’s Note

All claims expressed in this article are solely those of the authors and do not necessarily represent those of their affiliated organizations, or those of the publisher, the editors and the reviewers. Any product that may be evaluated in this article, or claim that may be made by its manufacturer, is not guaranteed or endorsed by the publisher.
